# Non-Alcoholic Fatty Pancreas Disease Pathogenesis: A Role for Developmental Programming and Altered Circadian Rhythms

**DOI:** 10.1371/journal.pone.0089505

**Published:** 2014-03-21

**Authors:** Rebeca Carter, Angelina Mouralidarane, Junpei Soeda, Shuvra Ray, Joaquim Pombo, Ruma Saraswati, Marco Novelli, Giuseppe Fusai, Francesca Rappa, Chiara Saracino, Valerio Pazienza, Lucilla Poston, Paul D. Taylor, Manlio Vinciguerra, Jude A. Oben

**Affiliations:** 1 Institute for Liver and Digestive Health, Royal Free Hospital, University College London, London, United Kingdom; 2 Department of Gastroenterology, Guy's and St Thomas' NHS Foundation Trust, London, United Kingdom; 3 Division of Women's Health, King's College London and King's Health Partners, London, United Kingdom; 4 Department of Pathology, University College London, London, United Kingdom; 5 Hepatobiliary and Liver Transplant Unit, Royal Free Hospital, University College London, London, United Kingdom; 6 Department of Experimental Biomedicine and Clinical Neurosciences, Section of Human Anatomy, University of Palermo, Palermo, Italy; 7 Euro-Mediterranean Institute of Science and Technology (IEMEST), Palermo, Italy; 8 Department of Medical Sciences, Gastroenterology Unit, IRCCS “Casa Sollievo della Sofferenza” Hospital, San Giovanni Rotondo, Italy; University of Verona, Ospedale Civile Maggiore, Italy

## Abstract

**Objectives:**

Emerging evidence suggests that maternal obesity (MO) predisposes offspring to obesity and the recently described non-alcoholic fatty pancreas disease (NAFPD) but involved mechanisms remain unclear. Using a pathophysiologically relevant murine model, we here investigated a role for the biological clock - molecular core circadian genes (CCG) in the generation of NAFPD.

**Design:**

Female C57BL6 mice were fed an obesogenic diet (OD) or standard chow (SC) for 6 weeks, prior to pregnancy and throughout gestation and lactation: resulting offspring were subsequently weaned onto either OD (Ob_Ob and Con_Ob) or standard chow (Ob_Con and Con_Con) for 6 months. Biochemical, pro-inflammatory and pro-fibrogenic markers associated with NAFPD were then evaluated and CCG mRNA expression in the pancreas determined.

**Results:**

Offspring of obese dams weaned on to OD (Ob_Ob) had significantly increased (p≤0.05): bodyweight, pancreatic triglycerides, macrovesicular pancreatic fatty-infiltration, and pancreatic mRNA expression of TNF-α, IL-6, α-SMA, TGF-β and increased collagen compared to offspring of control dams weaned on to control chow (Con_Con). Analyses of CCG expression demonstrated a phase shift in CLOCK (−4.818, p<0.01), REV-ERB-α (−1.4,p<0.05) and Per2 (3.27,p<0.05) in association with decreased amplitude in BMAL-1 (−0.914,p<0.05) and PER2 (1.18,p<0.005) in Ob_Ob compared to Con_Con. 2-way ANOVA revealed significant interaction between MO and post-weaning OD in expression of CLOCK (p<0.005), PER1 (p<0.005) and PER2 (p<0.05) whilst MO alone influenced the observed rhythmic variance in expression of all 5 measured CCG.

**Conclusions:**

Fetal and neonatal exposure to a maternal obesogenic environment interacts with a post-natal hyper-calorific environment to induce offspring NAFPD through mechanisms involving perturbations in CCG expression.

## Introduction

Non-alcoholic fatty pancreas disease (NAFPD) is a recently described disease entity associated with an obese and/or dysmetabolic phenotype [1], [2]. NAFPD describes a phenotype ranging from deposition of fat in the pancreas to pancreatic inflammation, and resultant fibrosis. This pancreatic phenotype is similar to that of obesity-induced liver disease, non-alcoholic fatty liver disease (NAFLD), which describes a spectrum from hepatic steatosis through steatohepatitis to cirrhosis, and possible hepatocellular carcinoma [2], [3]. Given that the liver and pancreas have similar embryological origins, it is also plausible, as suggested for NAFLD, that obesity may lead to pancreatic cancer through pancreatic steatosis [4], [5]. This hypothesis is corroborated by studies implicating NAFPD as a risk factor in pancreatic adenocarcinoma [6]–[8].

The prevalence of maternal obesity is increasing worldwide in parallel with adult obesity rates [9], and observational studies suggest an association between maternal obesity, and risk of childhood obesity [10], [11], for which rates are similarly rising with 30% of US and UK children, aged 2 to 15, now classed as overweight or obese [12], [13]. We have previously shown, in a rodent model, that diet induced maternal obesity can play a causative role in the development of NAFPD and that this is exacerbated if the offspring themselves are reared on the same obesogenic diet [1].

The concept of developmental programming suggests that the early environment from conception through to the early post-natal period can alter gene expression through epigenetic processes in the developing offspring, resulting in a permanent alteration in offspring physiology [14]–[16]. Nutrition is considered a ‘major intrauterine environmental factor that alters expression of the fetal genome’ [17], [18], but mechanistic pathways are unclear.

Circadian clocks are molecular oscillators, which drive daily rhythms of physiology and behaviour [19]. The molecular machinery encoding the biological ‘clock’ involves a transcriptional/translational negative feedback loop. The heterodimer CLOCK (circadian locomotor output kaput cycles) and BMAL1 (brain and muscle anrt-like 1) heterodimer complex is controlled, through a negative feedback loop involving Period and Cryptochrome genes [19]. Regulatory accessory pathways include REV-ERB-α, which modulates the circadian clock through BMAL1 expression [19], [20].

Homeostasis is achieved through interactions between the pace-setting hypothalamic suprachiasmatic nucleus (SCN), also known as the master clock, and the peripheral clocks, located in all body cells. The master clock acts as a pacesetter for all peripheral clocks and is predominantly entrained by light [21]. Peripheral clocks however, have been shown to be capable of acting autonomously, and are sensitive to changes in nutritional status [22]. An association between disruption of CCG and metabolism has been shown in several models [19], [21] and, specifically, is implicated in the regulation of metabolic processes executed by the pancreas, including islet cell growth and development [23].

Mice with *CLOCK* gene mutations are reported to be hyperphagic and obese [24], and a high fat diet has been reported to alter the expression and rhythmicity of CCG in rodents [25]. Recently, isolated reports have suggested that genes contributing to circadian ‘clocks’ may be vulnerable to modulation by the nutritional environment in early life [26], [27]. We therefore hypothesised that maternal over-nutrition may programme offspring dysmetabolism via an altered expression of CCG, resulting in a permanent disruption of circadian rhythms in the pancreas during development.

Specifically, we interrogated the potential for mechanistic involvement of CCG in the pathogenesis of NAFPD arising from an interaction between maternal obesity and a post-weaning nutritional status.

## Materials and Methods

All studies were approved by Local University College London Ethics Committee, and conducted under UK Home Office, Animal in Science Regulation Unit (Scientific Procedures) Act 1986 guidelines. Female C57BL/6J mice (n = 60), proven breeders (one previous litter) and approximately 100-day-old (Charles River Laboratories, UK) were maintained under controlled conditions (22°C, 12-hr light/dark cycle) and fed either a standard chow diet (RM1: 15% crude protein, 50% polysaccharides, 7% simple sugars, 5.3% fibre, 3% lipids, 0.2% methionine, 3.3 kcal/g) *ad libitum*. They were then randomly allocated to either a control standard chow (RM1, Special Dietary Services, UK) or a semi-synthetic energy-rich and highly palatable obesogenic diet (10% simple sugars, 20% animal lard 28% polysaccharide, 23% protein (w/w), Special Dietary Services, UK, energy 4.5 kcal/g). The pelleted obesogenic diet was supplemented with *ad libitum* access to sweetened condensed milk (approximately 55% simple sugar, 8% fat, 8% protein, w/w, Nestle, SZ) with added micronutrient mineral mix (AIN93G, Special Dietary Services, UK). Combined intake calculated from measured daily intake of pellets and milk (approximately 16% fat, 33% simple sugars, 15% protein, energy 4.0 kcal/g) [28]. After 6–8 weeks females on the obesogenic diet, achieved a 30% increase in body weight and were then mated with C57BL/6J males (day 0 pregnancy signified by the appearance of a copulation plug). Dams were maintained on the obesogenic or control diet throughout gestation and suckling. Following spontaneous delivery, dams and their litters were left undisturbed for 48 hours when litters were standardized to 6 pups with an equal number of males and females (3♂, 3♀), wherever possible, to standardise milk supply. At 3 weeks, offspring were weaned onto either the control (RM1, standard chow, SC, n = 4–5/group) or obesogenic diet (OD) (n = 4–5/group) until 6 months of age. Offspring had *ad libitum* access to food and water and were maintained in a 12-hour light/dark cycle in a thermostatically controlled environment (22°C). Each of the four groups included pups randomly selected from litters born to different dams.

### Offspring Husbandry

The allocation of maternal and offspring post-weaning diet provided 4 groups:

- maternal diet of SC followed by a post-weaning diet of SC (Con_Con, n = 4–5).
- maternal diet of SC followed by a post-weaning OD (Con_Ob, n = 4–5).
- an obesogenic maternal diet followed by a post-weaning diet of SC (Ob_Con, n = 4–5).
- an obesogenic maternal diet followed by a post-weaning obesogenic diet (Ob_Ob, n = 4–5).

### Glucose tolerance test

An oral glucose tolerance test (OGTT) was performed at 6 months, as previously described, with some modification [29]. Briefly mice were fasted for 5 h and D-glucose at 1.5 g/kg was orally administered. OGTT test was performed using different sets of mice, kept under the same experimental conditions as in the main experiments, to avoid the possible influence of fasting on circadian analyses [29].

### Tissue collection

At 6 months of age, offspring being maintained on a 12 h light/12 h dark cycle with food and water available *ad libitum*, were sacrificed at 4 hourly intervals over a 24-hour period, to allow analyses of CCG expression over a 24-hour period (Zeitgeber Time (ZT) 0, 4, 8, 12, 16, and 20, where ZT0 = light on and ZT12 = light off). Following sacrifice, blood samples were taken and harvested organs appropriately stored until analysed. For sample collection in the dark period, mice were transferred to a lit room and terminated within a few minutes of transfer.

### Fibrogenic markers and circadian genes

Pancreatic tissue mRNA was assayed by quantitative 2-step PCR for expression of pro-inflammatory markers (Interleukin-6 (IL-6) and tumour necrosis factor-α (TNFα), pro-fibrogenic markers, (collagen type 1-α2; α-smooth muscle actin, αSMA and transforming growth factor–β, TGF-β1) and CCG (*Clock, BMAL-1, Period 1, Period 2 and REVERB*- α) using pups from all time points. Primer sequences were as shown in Table 1. GAPDH was used as reference gene. The reference were determined by the software program geNORM (Primerdesign, UK).

**Table 1:** Primer sequences were obtained for the relevant genes of interest from GenBank (except for the primers for the circadian study arm).

**Collagen Type 1α**	Sense Primer: 5′-GAACGGTCCACGATTGCATG-3′ Antisense Primer: 5′-GGCATGTTGCTAGGCACGAAG-3′ Annealing temp: 55°C	Expected Weight: 167 bp
**TGF-ß1**	Sense Primer: 5′-AAAATCAAGTGTGGAGCAAC-3′ Antisense Primer: 5′-CCACGTGGAGTTTGTTATCT-3′ Annealing temp: 59°C	Expected Weight: 224
**αSMA**	Sense Primer: 5′-ATCTGGCACCACTCTTTCTA-3′ Antisense Primer: 5′-GTACGTCCAGAGGCATAGAG-3′ Annealing temp: 59°C	Expected Weight: 191 bp
**IL-6**	Sense Primer: 5′-GTTTGGTAGCATCCATCATT-3′ Antisense Primer: 5′-TTCACAGAGGATACCACTCC-3′ Annealing temp: 55°C	Expected Weight: 203 bp
**TNF-α**	Sense Primer: 5′-CCCTTCATCTTCCTCCTTAT-3′ Antisense Primer: 5′-TCCAGCTGACTAAACATCCT-3′ Annealing temp: 55°C	Expected Weight: 220 bp
**GAPDH**	Sense Primer: 5′-CACAATTTCCATCCCAGACC-3′ Antisense Primer: 5′-GGGTGCAGCGAACTTTATTG-3′ Annealing temp: 60°C	Expected Weight: 93 bp
**Clock**	Cat. No: QT00197547. Annealing temp: 55°C	Qiagen
**Period 1**	Cat. No: QT00113337. Annealing temp: 55°C	Qiagen
**Period 2**	Cat. No: QT00198366. Annealing temp: 55°C	Qiagen
**REV-ERB α**	Cat. No: QT00164556. Annealing temp: 55°C	Qiagen
**BMAL-1**	Cat. No: QT00101647. Annealing temp: 55°C	Qiagen

The primer sequences were analyzed for optimum primers using Primer 3 Software. Ready-to-use Quantitect Primer Assays (Qiagen) were purchased for the circadian arm of the study. Each assay contains forward and reverse primers that are generated from the NCBI Reference Sequence database, optimised and bioinformatically validated.

### Western Blotting

Pancreata from the four experimental mice groups were lysed and processed for immunoblotting analysis with specific CLOCK and BMAL1 antibodies as previously described [30], [31]. We pooled together (n = 4) and analyzed equal amounts of proteins lysates of pancreata for each group/time point, as previously reported [32].

### Tissue Triglyceride Content

An adaptation of the Folch Method and triglyceride assay reagents (Roche Diagnostics) [33] was used to determine murine whole pancreas tissue triglyceride content.

### Histology

Following sacrifice, sections of pancreas were fixed in formalin with the spleen attached to aid orientation in histological analysis. Sections were embedded in paraffin and stained with both H&E and Masson's Trichrome to determine adipocyte infiltration and pericellular fibrosis respectively. Fat infiltration and extent of fibrosis was graded as previously described [34].

### Statistical Analysis

Data are expressed as mean ± standard error of the mean (SEM). Means of each group were compared using both one-way and two-way ANOVA, as indicated. Statistical significance was assumed as p<0.05. Cosinor analysis was employed, in addition to ANOVA, to determine rhythmicity of circadian gene expression within a 24-hour period. Cosinor analysis evaluates the ‘mesor’ (circadian rhythm adjusted mean, based on the parameters of a cosine function), timing of the oscillatory crest and amplitude, p<0.05 regarded as significant. Two-way ANOVA was also used to determine the effect of gender and nutritional group on the observed variance between offspring and the relative influence of maternal verses postnatal diet on offspring phenotype. Since no main effect of gender was observed for the reported biomarkers (Table 2) male and female data were combined in the cosinor analysis. The statistical unit ‘n’ used throughout the analysis is number of dams, not number of pups. Statistical analysis was conducted using GraphPad Prism 5 and Stata version 11.2 (StataCorp, College Station, Texas).

**Table 2:** Two-Way ANOVA results when comparing the influence of gender and group on the overall variance seen between offspring.

	Variance Attributed By…		
Pancreatic Marker	Sex	Group	Overall Interaction
**IL-6**	No Significance	67.61% (p<0.0001)	No Significance
**TNF-α**	No Significance	85.52% (p<0.0001)	No Significance
**α-SMA**	No Significance	60.74% (p<0.0001)	No Significance
**Collagen**	No Significance	57.17% (p<0.0001)	No Significance
**TGF-β**	No Significance	53.90% (p<0.0007)	No Significance

## Results

### Dysmetabolic Phenotype and NAFPD

#### Body Weights

**Figure 1:**
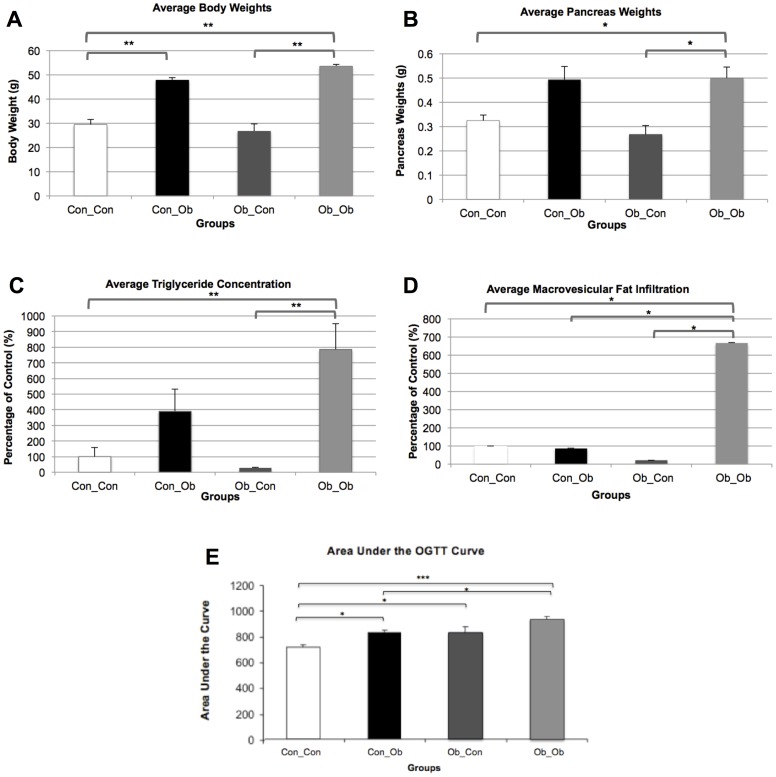
Anthropometric Data: Con_Con, Con_Ob, Ob_Con, Ob_Ob. A = Offspring Body Weights; B = Offspring Pancreas Weights; C = Pancreatic Triglyceride Concentrations; D = Macrovesicular Fat Infiltration; E = Oral Glucose Tolerance Test. * = p<.05; ** = p<.001; *** = p<.0001 (n = 4–5/group). ANOVA with Tukey post hoc test.

As described in detail above, the allocation of maternal and offspring post-weaning diets provided 4 groups: Con_Con; Con_Ob; Ob_Con; Ob_Ob. Offspring exposed to a post-weaning OD (Con_Ob and Ob_Ob) were significantly heavier than control (Con_Con; p<0.001; p<0.001). Two-way ANOVA further revealed 83% of the variance seen between the groups to be attributable to the post-weaning diet (p<0.0001), with no apparent main effect of MO on body weight, and no interaction between MO and post-weaning diet attributed to variance (Figure 1A).

#### Pancreas Weights

At 6 months, only offspring exposed to both interventions (Ob_Ob) were found to have significantly heavier pancreas weights compared to control (Con_Con, p<0.01). Moreover, these offspring also displayed heavier pancreata compared to offspring exposed to MO alone (Ob_Con, p<0.01). Two-way ANOVA attributed the post-weaning diet to 64.18% of the observed variance between the groups (p<0.001), with no statistical main effect of MO, nor an interaction between the variables as responsible for the variance (Figure 1B).

#### Triglyceride Content

As for pancreatic triglyceride content, this was significantly greater in Ob_Ob offspring than both Ob_Con offspring (p<0.001) and Con_Con offspring (p<0.001). There was no significant difference between offspring exposed to a post-weaning OD alone (Con_Ob) and control. Two-way ANOVA attributed 54.38% of the observed variance to the post-weaning diet (p<0.01) but no statistical power was attributed to the effect of MO (Figure 1C).

*Pancreatic Macrovesicular Fat Infiltration* was significantly greater in Ob_Ob compared to all other groups: Ob_Con (p<0.05), Con_Ob (p<0.05) and Con_Con (p<0.05) (Figure 1D, n = 4–5). Two-way ANOVA attributed 23% (p<0.05) of observed variance to a post-weaning diet, with no apparent main effect of MO. However, 24.61% (p<0.05) of observed variance was attributed to an interaction between MO and post-weaning OD on two-way ANOVA. (Figure 1D).

#### Oral Glucose Tolerance Test

The influence of maternal obesity on offspring glucose tolerance was evident following an oral glucose tolerance test (OGTT), with both Ob_Con offspring (p<0.05) and Ob_Ob offspring (p<0.0001) displaying a significant increase in AUC compared with Con_Con offspring (Figure 1E and Figure S1). Moreover, the OB_Ob AUC was significantly greater than the response of Con_Ob (p<0.05, Figure 1E), indicating the influence of maternal obesity on offspring glucose metabolism. An influence of the postnatal diet was also apparent with Con_Ob demonstrating a significant increase in AUC compared with Con_Con offspring (p<0.05).

#### Histology

**Figure 2:**
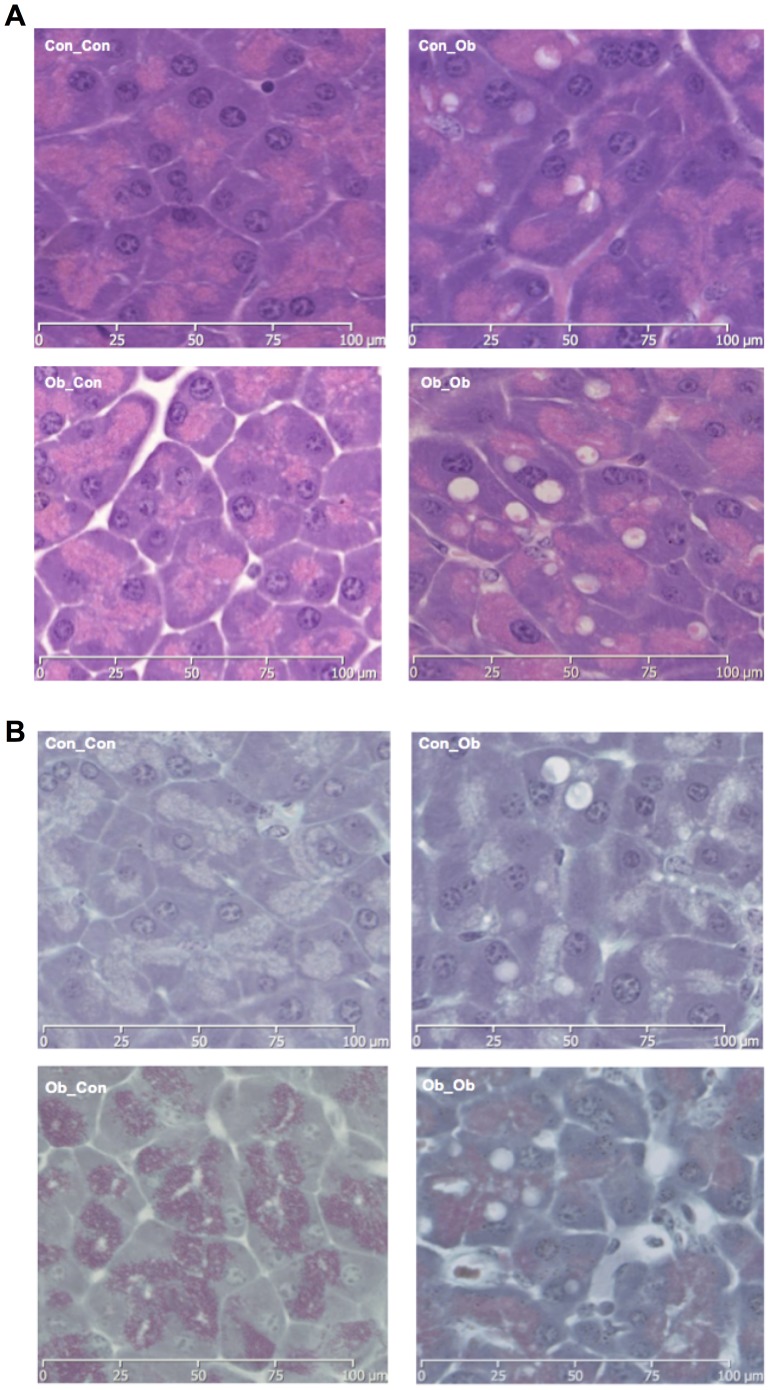
Pancreatic Histology: Con_Con = Top Left; Ob_Ob = Top Right; Ob_Con = Bottom Left; Ob_Ob = Bottom Right. A = Haematoxylin and Eosin Stain for Pancreas Histology (×40); B = Masson's Trichrome Stain for Pancreas Histology (×40) (n = 4–5/group).

Histological analysis (H&E staining) of pancreata revealed a marked increase in macrovesicular adipocyte infiltration in offspring exposed to both obesogenic interventions (i.e. Ob_Ob) (Figure 2A, Ob_Ob compared to the other groups), confirming the biochemical results above (Figure 1). Upon scoring for intra-lobular fat Ob_Ob offspring displayed higher values (0.73±0.02), compared to Con_Con (0.02±0.003), Con_Ob (0.2±0.01), or Ob_Con (0.3±0.02) [33]. Masson's Trichrome staining for pericellular fibrosis highlighted areas of occasional increased fibrosis in Ob_Ob offspring (Figure 2B, Ob_Ob compared to the other panels) but on scoring fibrosis grade was not significantly different between the groups.

### Pancreatic Inflammation and fibrosis

We then sought to analyze the gene expression levels of the major inflammatory cytokines (IL-6, TNF-α) and the fibrotic indicators (α-SMA, collagen, TGF-β) in the pancreata of the 4 mice groups, to test the potential effects of MO and post-weaning diets on these pathogenic processes. All the marker tested in PCR showed same trend across the gropus in all time points, therefore, ZT 8 was chosen for further analysis in all groups.

#### IL-6

**Figure 3:**
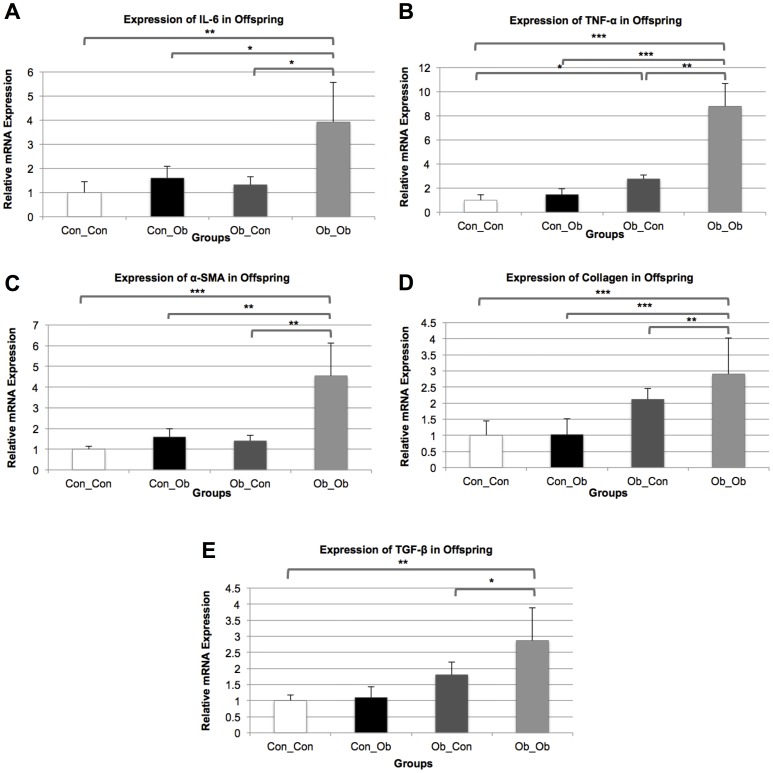
mRNA Expression of markers of Pancreatic Injury: Con_Con, Con_Ob, Ob_Con, Ob_Ob. A = IL-6; B = TNF-α; C = α-SMA; D = Collagen; E = TGF- β. * = p<.05; ** = p<.001; *** = p<.0001 (n = 4–5/group). ANOVA with Tukey post hoc test.

IL-6 mRNA expression in Ob_Ob offspring was higher when compared to all other groups: versus Ob_Con group (p<0.05), versus Con_Ob group (p<0.05), and versus Con_Con (p<0.001). There was no significant difference between offspring exposed to a post-weaning OD alone (Con_Ob) or MO alone (Ob_Con) compared to controls (Con_Con), indicating the need for the combined presence of both interventions to induce upregulation of IL-6 expression. Two-Way ANOVA further corroborated these results, revealing MO and the post-weaning OD to be attributable to 43.71% (p<0.001) and 34.53% (p<0.01) of the overall variance in IL-6 expression respectively, but also, that there was a significant interaction between these variables, contributing 10.18% (p<0.05) of the overall variance (Figure 3A).

#### TNF-α

The influence of the interventions on IL-6 was mirrored in TNF-α, another marker of pancreatic inflammation, with Ob_Ob offspring demonstrating a significantly increased expression of this cytokine compared to the other offspring groups: versus Ob_Con (p<0.001), versus Con_Ob (p<0.0001) and versus Con_Con (p<0.0001). In addition, MO was found to have an independent effect with Ob_Con offspring exhibiting significant upregulation of TNF-α compared with control (p<0.05). Two-way ANOVA attributed 61.22% and 18.35% of the overall variance to MO (p<0.0001) and the post-weaning diet to (p<0.001), respectively. An overall significant interaction between the variables attributed 4.63% (p<0.05) of the total variance. As with IL-6, there was no significant upregulation of TNF-α by a post-weaning OD in isolation, compared with control (Figure 3B).

### Markers of Pancreatic Fibrosis

#### α-SMA

The influence of MO on markers of offspring pancreatic fibrotic injury was evident in α-SMA expression, with Ob_Ob displaying upregulation compared to the other offspring groups: versus Ob_Con (p<0.001); versus Con_Ob (p<0.001) and versus Con_Con (p<0.0001). Two-way ANOVA further supported these results, attributing 63.77% (p<0.0001) and 13.08% (p<0.01) of the observed variance to MO and post-weaning OD respectively, and 5.53% (p<0.05) of the variance to an overall interaction between the variables. No significant difference was noted between offspring exposed to a post-weaning OD alone (Con_Ob) and control (Figure 3C).

#### Collagen

Similarly, Ob_Ob collagen mRNA expression was significantly greater than the other 3 offspring groups: versus Ob_Con (p<0.001), versus Con_Ob (p<0.0001), and versus Con_Con (p<0.0001). Two-way ANOVA attributed 61.26% (p<0.0001) and 28.39% (p<0.001) of observed variance to MO and post-weaning OD respectively and 7.14% (p<0.05) of the variance to an overall interaction between the variables. Again, no significant difference was observed between Con_Oband Con_Con (Figure 3D).

#### TGF-β

TGF-β mRNA expression was significantly greater in Ob_Ob compared with Ob_Con (p<0.05) and Con_Con (p<0.001) indicating an additive effect of the postweaning obesogenic diet. No significant difference was noted between Ob_Ob and Con_Ob, nor was a significant difference noted between Con_Ob and control. Two-way ANOVA revealed MO contributing 64% (p = 0.0009) of the observed variance, indicating MO as having the main effect on the observed variance in results (Figure 3E).

### Core Circadian Genes

**Figure 4:**
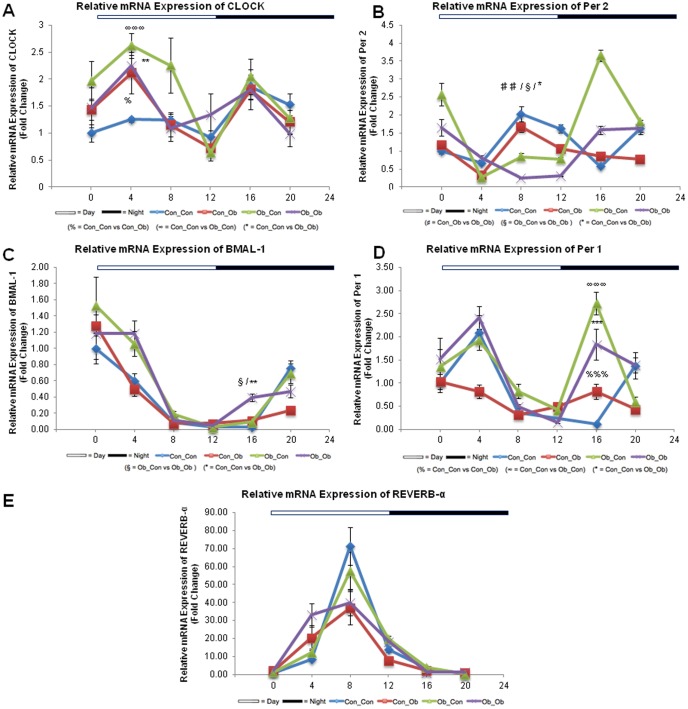
mRNA Expression of Circadian Genes: Con_Con, Con_Ob, Ob_Con, Ob_Ob. A = Clock; B = Per-2; C = BMAL-1; D = Per-1; E = REV-ERB-α. %%% = p<.0001; ∞∞∞ = p<.0001; * = p<.05; ** = p<.001; *** = p<.0001; ## = p<.001; § = p<.05. (% = Con_Con versus Con_Ob; ∞ = Con_Con versus Ob_Con; * = Con_Con versus Ob_Ob; # = Con_Ob versus Ob_Ob; § = Ob_Con versus Ob_Ob) (n = 4–5/group). ANOVA with Tukey post hoc test.

Results are divided into ANOVA and cosinor analysis of CCG of offspring over 6 separate time points (Figure 4):

### ANOVA Analysis of Circadian Gene mRNA Expression

Both CLOCK and Per2 gene expression displayed differences as a result of MO and a post-weaning OD. Analysis of CLOCK gene expression revealed a significant difference between Con_Con expression and the 3 other groups at ZT4: vs Ob_Ob (p<0.001), vs Ob_Con (p<0.0001) and vs Con_Ob (p<0.05). Two-way ANOVA revealed that MO accounted for 64% of the total variance seen and an interaction between the variables as accountable for 23% (p<0.05) (Figure 4A). Per-2 gene expression revealed a different pattern, with a decreased expression in Ob_Ob compared with the three other groups at ZT8: vs Ob_Con (p<0.05), vs Con_Ob (p<0.001) and vs Con_Con (p<0.05). Two-way ANOVA further revealed an overall interaction as being attributable to 23% of total variance (p<0.05), with MO attributing a further 39% (p<0.05), similarly indicating that MO had the main effect on the observed variance (Figure 4B).

At ZT16, a perturbation was also observed for BMAL-1 as noted for Per-2 expression at ZT8 (Ob_Ob vs all other groups, p<0.05). Two-way ANOVA revealed that MO and an overall interaction between MO and a post-weaning OD accounted for 18%, and 54% variance respectively, p<0.05. Additionally, the post-weaning diet contributed a further 44% (p<0.05) of the overall variance (Figure 4C).

Further interactions between the sequential circadian genes were observed with the same patterns of gene expression noted for CLOCK at ZT4 and Per-1 at ZT16. As observed with CLOCK at ZT4, Per-1 gene expression was different between Con_Con and Con_Ob (p<0.0001), Ob_Con (p<0.0001) and Ob_Ob (p<0.0001). Two-way ANOVA revealed an overall interaction between MO and OD as contributing 10% of total variance (p<0.05), MO alone contributing 49% (p<0.0001) and the post-weaning OD 24% (p<0.001), (Figure 4D). Two-way ANOVA revealed that MO attributed to 31% (p<0.05) of the variance in gene expression.

### Protein levels of core clock genes CLOCK and BMAL1

To determine if variations observed at the mRNA level were mirrored at the protein level, we now analyzed protein expression levels of the core components of the circadian clock machinery, CLOCK and BMAL1, in the pancreata of the 4 experimental groups at ZT 0 h, 8 h and 16 h by immunoblotting (Figure S2). Strikingly higher protein levels of CLOCK and BMAL1 were observed at ZT16, with a peak of BMAL1 expression present in the Ob_Ob offspring, while an increased expression of CLOCK was detected in the Con_Ob group. Highest levels of CLOCK and BMAL1 proteins at ZT16 are consistent with observed mRNA expression (Figure 4A and C).

### Cosinor Analysis of mRNA Expression

#### Clock

**Figure 5:**
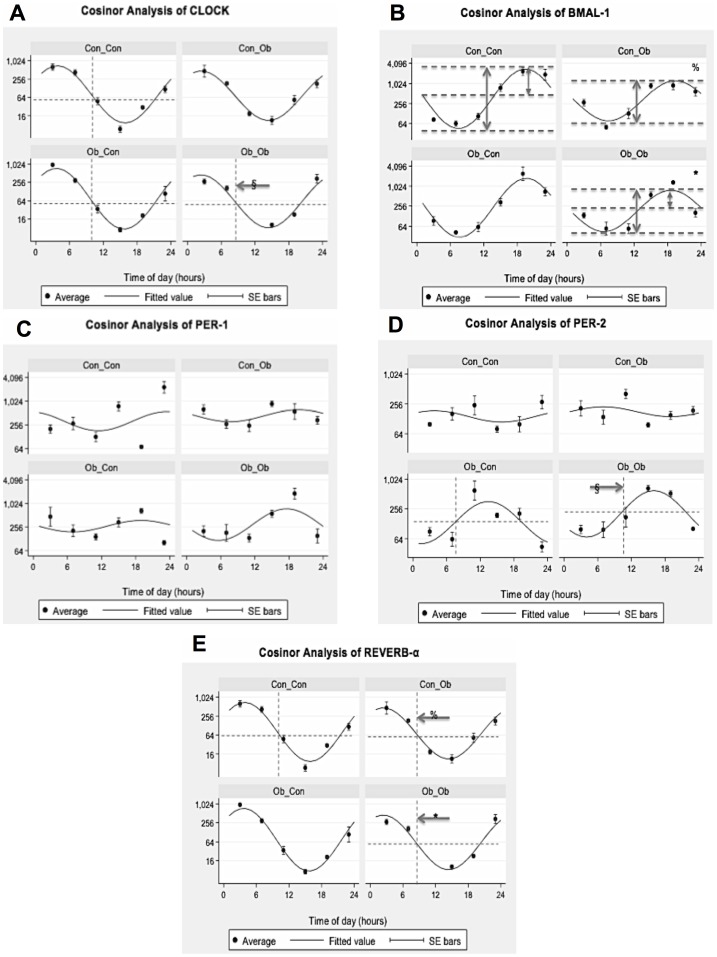
Cosinor Analysis of Circadian Genes: Con_Con, Con_Ob, Ob_Con, Ob_Ob. A = Clock; B = Bmal-1; C = Per-1; D = Per-2; E = REV-ERB-α. (% = Con_Con versus Con_Ob; * = Con_Con versus Ob_Ob; § = Ob_Con versus Ob_Ob) (n = 4–5/group).

Cosinor analysis revealed a phase shift of −4.818005 Hours (p<0.01) when comparing Ob_Ob with Ob_Con, suggesting a main effect of the postweaning diet. No other significant differences were seen between the groups in relation to CLOCK gene expression (Figure 5A; Table 3).

**Table 3:** Results of Cosinor Analysis of Circadian Gene mRNA Expression.

Gene of Interest	Significant Difference			
	Phase	Amplitude	Maxima	Minima
**CLOCK**	Ob_Con vs Ob_Ob (p = 0.009)	No	No	No
				
**BMAL 1**	No	Con_Con vs Con_Ob (p = 0.016)	Con_Con vs Con_Ob (p = 0.013)	No
		Con_Con vs Ob_Ob (p = 0.028)		
**Per 1**	No	No	No	No
				
**Per 2**	Ob_Con vs Ob_Ob (p = 0.026)	Con_Con vs Ob_Con (p = 0.017)	Con_Con vs Con_Ob (p = 0.013)	Con_Con vs Ob_Ob (p = 0.002)
		Con_Con vs Ob_Ob (p = 0.005)		
**REV-ERB α**	Con_Con vs Con_Ob (p = 0.002)	No	No	No
	Con_Con vs Ob_Ob (p = 0.002)			

#### BMAL-1

Cosinor analysis revealed significantly reduced amplitude, with respect to Con_Con, in Ob_Ob offspring (−0.914, p<0.05) and Con_Ob offspring (−0.972, p<0.05), suggesting main effect of postweaning diet. Furthermore, maxima were calculated to be significantly reduced in Ob_Ob offspring when compared with Con_Con (−1.86 p<0.05). No other significant differences were seen between the groups in relation to BMAL-1 gene expression (Figure 5B; Table 3).

#### Period 1

Cosinor analysis yielded no statistically significant difference between the groups in Per 1 (Figure 5C; Table 3).

#### Period 2

There was a phase shift of 3.27 Hours (p<0.05) between Ob_Ob and Ob_Con offspring suggesting main effect of MO. Amplitude was also calculated as increased in Ob_Ob (1.03, p<0.05) and Ob_Con (1.18, p<0.005), when compared with Con_Con suggesting evidence of both MO and an interaction between the variables as having a significant effect. Furthermore, maxima were increased in Ob_Ob compared with Con_Con (1.68, p<0.05), and minima decreased Ob_Con compared with Con_Con (−1.11, p<0.05). No other significant differences were seen between the groups in relation to Per-2 gene expression (Figure 5D; Table 3).

#### REVERB-α

There was a phase shift of −1.4 Hours (p<0.05) when comparing offspring exposed to a post-weaning OD (Con_Ob, Ob_Ob) with COn_Con offspring. No other significant differences were seen between the groups in relation to REVERB-α gene expression (Figure 5E; Table 3).

## Discussion

NAFPD is an emerging clinical entity associated with dysmetabolism and characterized by pancreatic fat deposition, inflammation, fibrosis and pancreatitis. This pancreatic phenotype is strikingly similar to that of obesity-induced NAFLD, which describes a spectrum characterized by hepatic steatosis, steatohepatitis and cirrhosis. The similarity in phenotypes could be due to the common embryonic origins of the liver and pancreas. We have previously shown that offspring exposure to MO throughout pregnancy and lactation induces a significant increase in markers indicative of a NAFPD phenotype, when compared to offspring exposed to a normal intrauterine and perinatal environment [1]. Here, we corroborate these results using a more pathophysiologically relevant model not involving cross-fostering which has since been shown to influence metabolic phenotype [35]. In addition to the previous findings of increased pancreatic triglycerides, collagen and TGF-β expression [1], our results show that MO in conjunction with a post-weaning OD (Ob_Ob) significantly impacts upon pancreas weight, pancreatic triglycerides concentration and macrovesicular fat concentration.

Moreover, offspring glucose tolerance was impaired not only in Ob_Ob but also in offspring exposed to MO in isolation (Ob_Con), accentuating an independent effect of MO. Thus, MO appears to have a deleterious effect on offspring phenotype, particularly when coupled with a post-weaning OD, which in isolation only significantly influenced offspring bodyweight. Since the offspring of obese mothers, weaned onto an OD (Ob_Ob) demonstrated the highest pancreatic triglyceride (TG) concentration, with no change in TGs in Con_Ob, this was most likely due to an interaction between the two interventions (maternal and post weaning OD diet) rather than the post-weaning diet alone (Figure 1C). The TG profile was mirrored in the infiltration of macrovesicular fat.

However, as Ob_Ob fat infiltration was higher than Con_Ob, this exposed an independent influence of MO in these offspring (Figure 1D). Thus, MO appears to have a priming or ‘first hit’ effect, which only become evident when offspring are subsequently challenged by a hypercalorific diet, exacerbating disease phenotype as a result. OGTT observations indicated impaired glucose tolerance in offspring of obese dams, which builds on a recent report [36], demonstrating that diet-induced obesity in female dams, whose offspring were weaned onto standard chow, led to insulin resistance at 8–11 months. Here we show this occurs at 6 months, and that it is exacerbated by a post-weaning obesogenic diet (Figure 1E).

Moreover, we found that control offspring exposed to a post-weaning OD showed less evidence of inflammation than offspring of the obese dams, on the same diet adding to evidence for a priming effect of MO. The higher TNF-α expression in the MO offspring, strengthens a role for MO in the programming of offspring disease. This is not the first study to demonstrate a role for MO in activation of inflammatory cytokines in offspring [1], [37], but is the first to demonstrate it in pancreatic tissue, in the absence of cross-fostering. Similar patterns were observed in markers of pancreatic fibrosis, further supporting the priming effect of MO. To summarize, these phenotypic results consistently highlight the importance of the maternal environment on offspring phenotype. This is emphasised by the two-way ANOVA analysis, which consistently implicate MO in the variance amongst the markers of pancreatic injury.

Support for the concept of programming of clock genes by nutrition is provided by a recent report in which adult offspring of protein-restricted rat dams demonstrated permanently altered expression in a functional network of hypothalamic nuclear receptors and co-regulators of the circadian clock involved in lipid metabolism [38]. Moreover, rats exposed to chronic phase shifts of the photoperiod during gestation showed altered energy balance with lifelong consequences for the metabolic homeostasis in the offspring which develop increased obesity and abnormal glucose metabolism [39].

Our results further report a significant disruption in CCG, which could have a mechanistic role for CCG in developmentally programmed NAFPD. Moreover, analysis of CCG expression at different stages of daylight revealed corresponding genes being affected in the same pattern but at opposite times of day (CLOCK and Per-2, BMAL-1 and Per-1; Figure 4A–D). We were able to confirm high protein expression levels of core clock component CLOCK and BMAL1 at ZT16, consistent with their mRNA levels (Figure S2). In addition, two-way ANOVA demonstrated a consistent role both for MO, and an interaction between MO and a post-weaning OD, in the observed variance in CCG. Cosinor analysis build on these observations, describing an array of perturbations in the normal diurnal rhythms, with differences predominantly found between Ob_Ob offspring and Con_Con offspring.

Overall, this evidence suggests that MO, in conjunction with a post-weaning OD, results in a significant disruption of the biological diurnal rhythms. Moreover, as no significant difference is seen between Con_Ob and Con_Con in isolation, in any of the CCG, further credence is given to role of MO in the perturbation of diurnal rhythms. As disruption of CCG has been implicated in altered regulation of metabolic pancreatic processes [40] and in hyperphagia and obesity [24], and the circadian rhythms are programmable [38], it is plausible that expression of CCG could be irreversibly altered following exposure to MO in utero. Thus, perturbation of CCG may be involved mechanistically in the priming effects of MO through epigenetic mechanisms, when the offspring is challenged post weaning with an OD.

In conclusion, our results, in a novel and pathophysiologically relevant NAFPD model, consistently show that offspring exposed to both MO and a post-weaning OD, have a more pronounced dysmetabolic and NAFPD phenotype than control since offspring exposed to a post-weaning OD in isolation were rarely significantly different to control and suggests, that MO has a ‘priming’ effect on offspring phenotype which in the presence of a post-weaning OD results in an exacerbated dysmetabolic offspring phenotype. These findings support the proposal that MO, through developmental programming, may be exacerbating the obesity epidemic and its associated metabolic disorders such as NAFPD [41]. Mechanistically, since CCG are entrainable by nutritional stimuli [19], [21], [41], [42], and in light of the significant differences between Con_Con and Ob_Con/Ob_Ob on cosinor and ANOVA analyses, we propose that the intra-uterine period may through perturbation of CCG be involved in programmed NAFPD.

## Supporting Information

Figure S1 **Oral glucose tolerance tests at 6 months.** Blood glucose concentrations, after oral glucose administration (1.5 g/kg), are represented. Ob_Ob showed significantly higher glucose levels at 15 minutes than Con_Con and Con_Ob (p<0.001 and p<0.05, respectively). **p<0.001 vs. Con_Ob, # p<0.05 vs. Con_Con.
(TIF)

Figure S2 Upper panel: pancreatic tissues from 4 different animals per group/time points were pooled together as previously described [32], lysed and equal amounts of proteins (45 µg) were loaded on a 10% polyacrylamide gel, separated by electrophoresis and immunoblotted with specific CLOCK and BMAL1 primary antibodies. β-actin expression served as loading control. Lower panel: densitometric quantification of BMAL1 and CLOCK proteins normalized to β-actin expression.
(TIF)
