# Supplementary figures and images for: Non-Alcoholic Fatty Pancreas Disease Pathogenesis: A Role for Developmental Programming and Altered Circadian Rhythms
Source: PLoS One. 2014 Mar 21;9(3):e89505. doi: 10.1371/journal.pone.0089505 (PMC3962337; doi:10.1371/journal.pone.0089505)

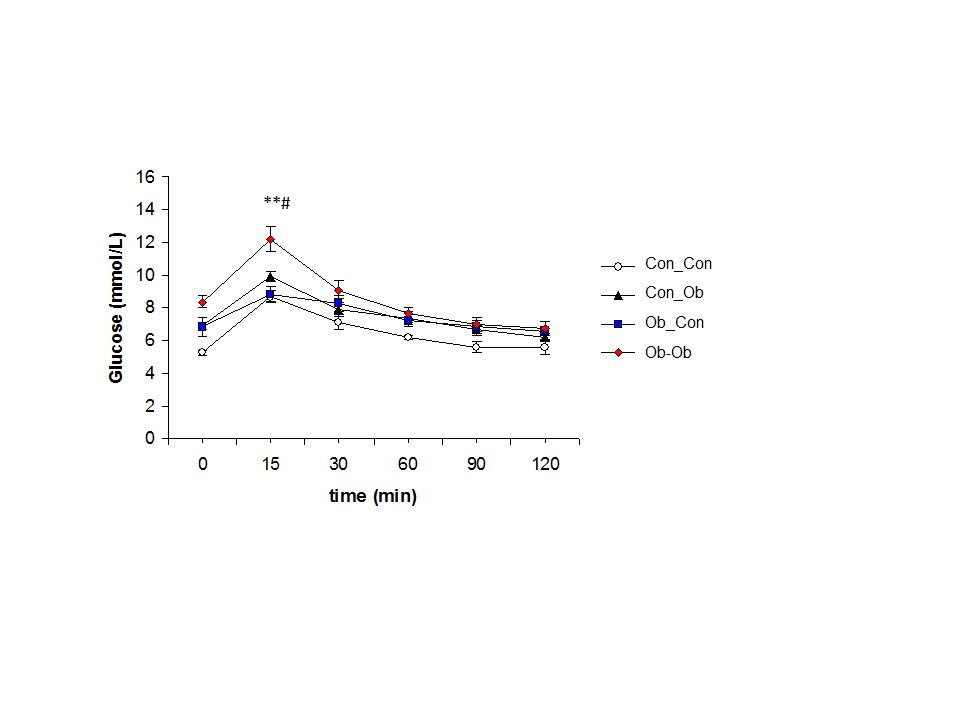

Supplement: Figure S1 — Oral glucose tolerance tests at 6 months. Blood glucose concentrations, after oral glucose administration (1.5 g/kg), are represented. Ob_Ob showed significantly higher glucose levels at 15 minutes than Con_Con and Con_Ob (p<0.001 and p<0.05, respectively). **p<0.001 vs. Con_Ob, # p<0.05 vs. Con_Con. (TIF) [file pone.0089505.s001.tif]

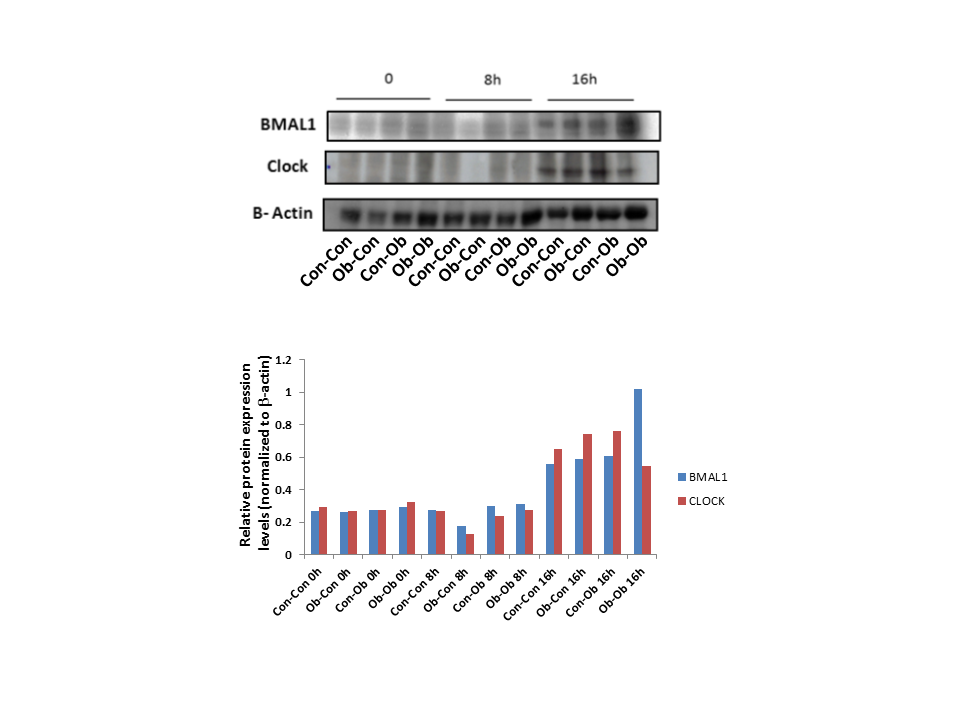

Supplement: Figure S2 — Upper panel: pancreatic tissues from 4 different animals per group/time points were pooled together as previously described [32], lysed and equal amounts of proteins (45 µg) were loaded on a 10% polyacrylamide gel, separated by electrophoresis and immunoblotted with specific CLOCK and BMAL1 primary antibodies. β-actin expression served as loading control. Lower panel: densitometric quantification of BMAL1 and CLOCK proteins normalized to β-actin expression. (TIF) [file pone.0089505.s002.tif]
